# (*R*)-[(*R*)-3-Benzyl-2-oxooxazolidin-4-yl][4-(methyl­sulfon­yl)phen­yl]methyl acetate

**DOI:** 10.1107/S1600536814009106

**Published:** 2014-04-26

**Authors:** Feng Li, Ming-Zhong Zhao, Chun-Hua Jin, Jian-Wei Zou

**Affiliations:** a School of Biological & Chemical Engineering, Ningbo Institute of Technology, Zhejiang University, Ningbo 315100, People’s Republic of China; b Ningbo Ocean & Fishery Bureau, Ningbo 315100, People’s Republic of China

## Abstract

The structure of the title compound, C_20_H_21_NO_6_S, is of inter­est with respect to its anti­bacterial properties. The oxazolidine ring makes dihedral angles of 79.63 (14) and 56.16 (12)° with the phenyl and benzene rings, respectively, while the phenyl and benzene rings make a dihedral angle of 64.37 (13)°. In the crystal, non-classical C—H⋯O hydrogen bonds link adjacent mol­ecules along the *c* axis.

## Related literature   

For the original synthesis of the title compound, see: Li *et al.* (2011 ▶). For inversion of the configuration of the sulfonyloxy moiety, see: Shi *et al.* (2010 ▶). For background to the anti-bacterial properties of thia­mphenicol-like compounds, see: Nagabhushan (1980 ▶, 1981 ▶); Jommi *et al.* (1985 ▶).
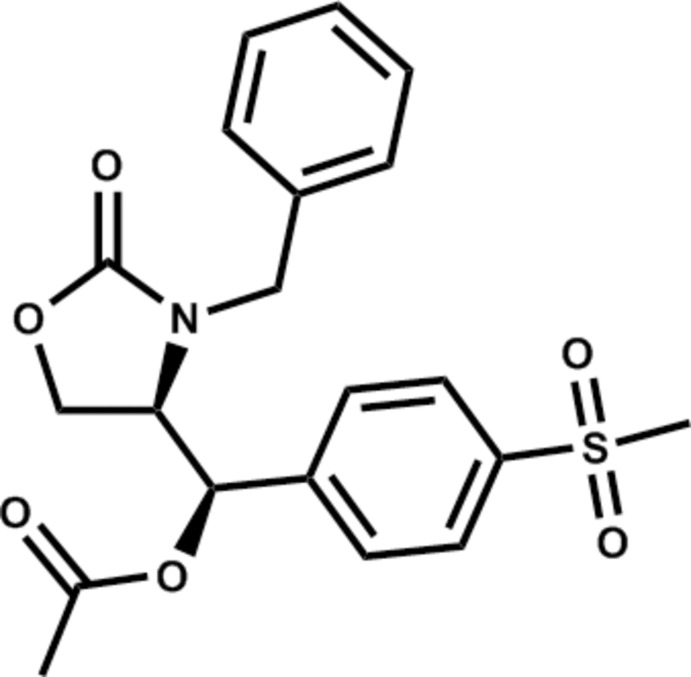


## Experimental   

### 

#### Crystal data  

C_20_H_21_NO_6_S
*M* *_r_* = 403.44
Monoclinic, 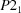

*a* = 5.837 (3) Å
*b* = 21.021 (10) Å
*c* = 7.884 (4) Å
β = 100.256 (7)°
*V* = 952.0 (7) Å^3^
*Z* = 2
Mo *K*α radiation
μ = 0.21 mm^−1^
*T* = 296 K
0.30 × 0.25 × 0.16 mm

#### Data collection  

Bruker SMART CCD area-detector diffractometer
Absorption correction: multi-scan (*SADABS*; Bruker, 2000 ▶) *T*_min_ = 0.940, *T*_max_ = 0.968
6495 measured reflections
3658 independent reflections
3254 reflections with *I* > 2σ(*I*)
*R*_int_ = 0.022

#### Refinement  

*R*[*F* ^2^ > 2σ(*F* ^2^)] = 0.035
*wR*(*F* ^2^) = 0.091
*S* = 1.02
3658 reflections
255 parameters
1 restraint
H-atom parameters constrained
Δρ_max_ = 0.14 e Å^−3^
Δρ_min_ = −0.17 e Å^−3^
Absolute structure: Flack (1983 ▶), 1356 Friedel pairs
Absolute structure parameter: −0.06 (6)

### 

Data collection: *SMART* (Bruker, 2000 ▶); cell refinement: *SAINT* (Bruker, 2000 ▶); data reduction: *SAINT*; program(s) used to solve structure: *SHELXTL* (Sheldrick, 2008 ▶); program(s) used to refine structure: *SHELXTL*; molecular graphics: *SHELXTL*; software used to prepare material for publication: *SHELXTL*.

## Supplementary Material

Crystal structure: contains datablock(s) I. DOI: 10.1107/S1600536814009106/hg5390sup1.cif

Structure factors: contains datablock(s) I. DOI: 10.1107/S1600536814009106/hg5390Isup2.hkl

Supporting information file. DOI: 10.1107/S1600536814009106/hg5390Isup3.cdx

Supporting information file. DOI: 10.1107/S1600536814009106/hg5390Isup4.cml

CCDC reference: 998863

Additional supporting information:  crystallographic information; 3D view; checkCIF report

## Figures and Tables

**Table 1:** Hydrogen-bond geometry (Å, °)

*D*—H⋯*A*	*D*—H	H⋯*A*	*D*⋯*A*	*D*—H⋯*A*
C7—H7*A*⋯O4^i^	0.97	2.52	3.373 (3)	147
C10—H10⋯O6^i^	0.98	2.54	3.384 (3)	144
C13—H13*C*⋯O1^ii^	0.96	2.55	3.305 (3)	135

Symmetry codes: (i) 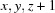; (ii) 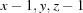.

**Fig. 1.**
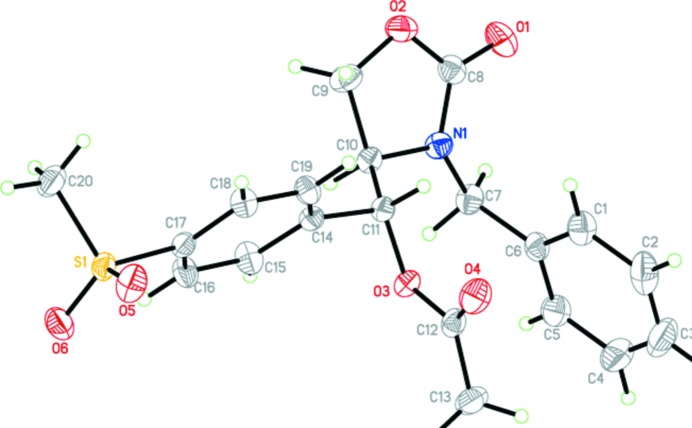
The ORTEP view of the title compound with 30% probability level ellipsoids.

**Fig. 2.**
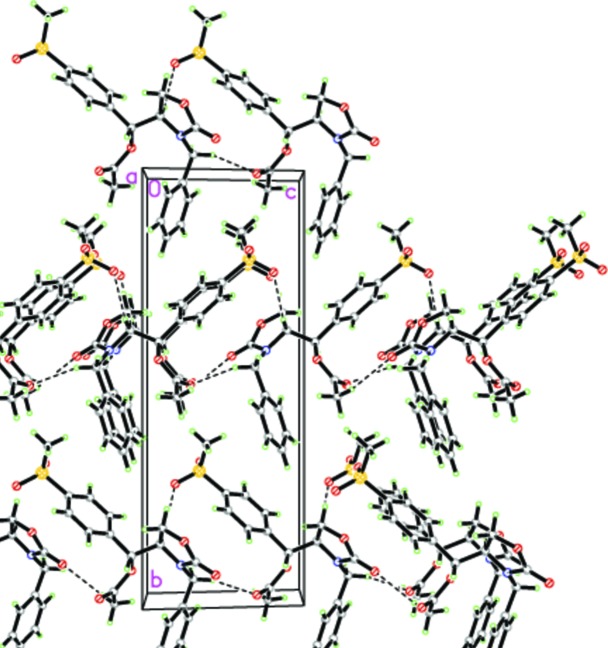
The molecular packing diagram of the title compound.

## supplementary crystallographic information

### 1. Comment

During the study on the synthesis of florfenicol, a class of anti­biotics with pronounced broad-spectrum anti­bacterial activity, (Nagabhushan, 1980 & Jommi *et al.*, 1985). the title compound was produced and is a key inter­mediate in the synthetic route to florfenicol (Li *et al.*, 2011). The title compound was synthesized through the nucleophilic substitution reaction of (*S*)-((*R*)-3-benzyl-2-oxooxazolidin-4-yl)(4-(methyl­sulfonyl) phenyl)­methyl­methane sulfonate. Here we report the crystal structure of the title compound.

Fig. 1 shows the molecular structure of the title compound. The enanti­omer was selected on the basis of the configuration of the starting material. All chiral carbon atoms (C10 and C11) are *R*-configuration. Only one molecule is included in the asymmetrical unit of this compound (Fig. 1). All the bond lengths and relevant angles are in the typical ranges. Although there is no –NH or –OH group available in the structure to form strong hydrogen bonds, the C atoms are involved in the formation of non-classical inter-molecular C—H···O hydrogen bonds (Fig 2).

### 2. Experimental

The literature procedure according to Li *et al.* (2011) was followed. A solution of 1,8-di­aza­bicyclo­[5.4.0]undec-7-ene (700 mg, 4.56 mmol) and glacial acetic acid (550 mg, 9.11 mmol) in anhydrous toluene (5 mL) was stirred for 1.5 h at room temperature. (*S*)-((*R*)-3-benzyl-2-oxooxazolidin-4-yl)(4-(methyl­sulfonyl)­phenyl)­methyl­methane­sulfonate was added and the reaction mixture was heated to 363 K for 8 h. The resulting mixture was cooled to r.t., diluted with CH_2_Cl_2_ (40 mL) and washed with 2M aq. HCl (30 mL), 10% aq. K_2_CO_3_ (30 mL), and brine (30 mL) successively. The organic phase was dried over Na_2_SO_4_, concentrated in vacuo. The residue was purified by flash chromatography to afford the title compound 850 mg (92%) as a white solid. Suitable crystals for X-ray experiments were obtained by slow evaporation from an AcOEt/CHCl_3_ solution at room temperature.

### 2.1. Refinement

Hydrogen atoms bonded to the carbon atoms were placed in calculated positions and refined as riding mode, with C—H = 0.93Å (methane) or 0.96Å (methyl) and U_iso_(H) = 1.2U_eq_ (C_methane_) or U_iso_(H) = 1.5U_eq_ (C_methyl_).

### Crystal data

**Table:**

C_20_H_21_NO_6_S	*F*(000) = 424
*M**_r_* = 403.44	*D*_x_ = 1.407 Mg m^−^^3^
Monoclinic, *P*2_1_	Mo *K*α radiation, λ = 0.71073 Å
Hall symbol: P 2yb	Cell parameters from 2349 reflections
*a* = 5.837 (3) Å	θ = 2.6–25.3°
*b* = 21.021 (10) Å	µ = 0.21 mm^−^^1^
*c* = 7.884 (4) Å	*T* = 296 K
β = 100.256 (7)°	Block, colourless
*V* = 952.0 (7) Å^3^	0.30 × 0.25 × 0.16 mm
*Z* = 2	

### Data collection

**Table:**

Bruker SMART CCD area-detector diffractometer	3658 independent reflections
Radiation source: fine-focus sealed tube	3254 reflections with *I* > 2σ(*I*)
Graphite monochromator	*R*_int_ = 0.022
phi and ω scans	θ_max_ = 27.8°, θ_min_ = 1.9°
Absorption correction: multi-scan (*SADABS*; Bruker, 2000)	*h* = −7→7
*T*_min_ = 0.940, *T*_max_ = 0.968	*k* = −19→27
6495 measured reflections	*l* = −10→9

### Refinement

**Table:**

Refinement on *F*^2^	Secondary atom site location: difference Fourier map
Least-squares matrix: full	Hydrogen site location: inferred from neighbouring sites
*R*[*F*^2^ > 2σ(*F*^2^)] = 0.035	H-atom parameters constrained
*wR*(*F*^2^) = 0.091	*w* = 1/[σ^2^(*F*_o_^2^) + (0.0516*P*)^2^] where *P* = (*F*_o_^2^ + 2*F*_c_^2^)/3
*S* = 1.02	(Δ/σ)_max_ < 0.001
3658 reflections	Δρ_max_ = 0.14 e Å^−^^3^
255 parameters	Δρ_min_ = −0.17 e Å^−^^3^
1 restraint	Absolute structure: Flack (1983), 1356 Friedel pairs
Primary atom site location: structure-invariant direct methods	Absolute structure parameter: −0.06 (6)

### Special details

**Table:**

Geometry. All e.s.d.'s (except the e.s.d. in the dihedral angle between two l.s. planes) are estimated using the full covariance matrix. The cell e.s.d.'s are taken into account individually in the estimation of e.s.d.'s in distances, angles and torsion angles; correlations between e.s.d.'s in cell parameters are only used when they are defined by crystal symmetry. An approximate (isotropic) treatment of cell e.s.d.'s is used for estimating e.s.d.'s involving l.s. planes.
Refinement. Refinement of *F*^2^ against ALL reflections. The weighted *R*-factor *wR* and goodness of fit *S* are based on *F*^2^, conventional *R*-factors *R* are based on *F*, with *F* set to zero for negative *F*^2^. The threshold expression of *F*^2^ > σ(*F*^2^) is used only for calculating *R*-factors(gt) *etc*. and is not relevant to the choice of reflections for refinement. *R*-factors based on *F*^2^ are statistically about twice as large as those based on *F*, and *R*- factors based on ALL data will be even larger.

### Fractional atomic coordinates and isotropic or equivalent isotropic displacement parameters (Å^2^)

**Table:**

	*x*	*y*	*z*	*U*_iso_*/*U*_eq_	
C1	0.2302 (4)	1.05248 (14)	1.2707 (4)	0.0558 (6)	
H1	0.1259	1.0324	1.3295	0.067*	
C2	0.1916 (6)	1.11380 (17)	1.2191 (5)	0.0781 (10)	
H2	0.0605	1.1349	1.2422	0.094*	
C3	0.3431 (7)	1.14521 (16)	1.1331 (5)	0.0833 (11)	
H3	0.3155	1.1872	1.0981	0.100*	
C4	0.5382 (6)	1.11300 (16)	1.0994 (4)	0.0701 (9)	
H4	0.6428	1.1335	1.0417	0.084*	
C5	0.5769 (4)	1.05031 (13)	1.1517 (3)	0.0506 (6)	
H5	0.7077	1.0290	1.1290	0.061*	
C6	0.4227 (4)	1.01946 (11)	1.2373 (3)	0.0395 (5)	
C7	0.4569 (4)	0.95231 (11)	1.3036 (3)	0.0418 (5)	
H7A	0.5095	0.9540	1.4274	0.050*	
H7B	0.3070	0.9311	1.2832	0.050*	
C8	0.8331 (4)	0.90008 (12)	1.3230 (3)	0.0418 (5)	
C9	0.7678 (4)	0.82331 (14)	1.1163 (3)	0.0491 (6)	
H9A	0.7290	0.7800	1.1428	0.059*	
H9B	0.8324	0.8231	1.0112	0.059*	
C10	0.5504 (3)	0.86602 (11)	1.0957 (3)	0.0347 (4)	
H10	0.4169	0.8418	1.1206	0.042*	
C11	0.4959 (3)	0.89357 (10)	0.9143 (2)	0.0325 (4)	
H11	0.6286	0.9188	0.8923	0.039*	
C12	0.2825 (4)	0.98383 (11)	0.7947 (3)	0.0401 (5)	
C13	0.0631 (5)	1.02073 (14)	0.7899 (3)	0.0567 (7)	
H13A	0.0890	1.0643	0.7625	0.085*	
H13B	0.0163	1.0185	0.9005	0.085*	
H13C	−0.0571	1.0031	0.7037	0.085*	
C14	0.4399 (3)	0.84297 (10)	0.7772 (2)	0.0329 (4)	
C15	0.2402 (4)	0.80602 (13)	0.7690 (3)	0.0453 (6)	
H15	0.1481	0.8107	0.8531	0.054*	
C16	0.1773 (4)	0.76262 (12)	0.6378 (3)	0.0435 (5)	
H16	0.0425	0.7386	0.6324	0.052*	
C17	0.3164 (3)	0.75514 (10)	0.5144 (2)	0.0343 (4)	
C18	0.5208 (4)	0.78958 (12)	0.5248 (3)	0.0394 (5)	
H18	0.6177	0.7831	0.4447	0.047*	
C19	0.5795 (3)	0.83348 (11)	0.6545 (3)	0.0380 (5)	
H19	0.7151	0.8572	0.6600	0.046*	
C20	0.4084 (5)	0.63552 (13)	0.3899 (3)	0.0563 (6)	
H20A	0.3814	0.6170	0.4959	0.084*	
H20B	0.5685	0.6483	0.4022	0.084*	
H20C	0.3739	0.6048	0.2988	0.084*	
N1	0.6212 (3)	0.91344 (9)	1.2290 (2)	0.0373 (4)	
O1	0.9319 (3)	0.92759 (11)	1.4488 (2)	0.0607 (5)	
O2	0.9295 (3)	0.84974 (9)	1.2545 (2)	0.0530 (4)	
O3	0.2955 (2)	0.93443 (7)	0.90796 (18)	0.0374 (3)	
O4	0.4311 (3)	0.99426 (10)	0.7128 (2)	0.0566 (5)	
O5	−0.0080 (3)	0.68428 (10)	0.3436 (2)	0.0585 (5)	
O6	0.2809 (3)	0.73099 (10)	0.1869 (2)	0.0574 (5)	
S1	0.22845 (9)	0.70219 (3)	0.34050 (6)	0.03925 (14)	

### Atomic displacement parameters (Å^2^)

**Table:**

	*U*^11^	*U*^22^	*U*^33^	*U*^12^	*U*^13^	*U*^23^
C1	0.0543 (14)	0.0493 (16)	0.0618 (15)	0.0027 (12)	0.0052 (11)	−0.0131 (13)
C2	0.078 (2)	0.0510 (19)	0.098 (2)	0.0168 (16)	−0.0056 (18)	−0.0156 (19)
C3	0.121 (3)	0.0371 (17)	0.078 (2)	0.0108 (18)	−0.020 (2)	0.0006 (15)
C4	0.105 (2)	0.0517 (18)	0.0496 (15)	−0.0264 (18)	0.0039 (15)	0.0018 (13)
C5	0.0619 (14)	0.0468 (15)	0.0438 (12)	−0.0066 (12)	0.0115 (11)	0.0000 (11)
C6	0.0452 (11)	0.0367 (12)	0.0348 (10)	−0.0035 (9)	0.0024 (8)	−0.0088 (9)
C7	0.0467 (11)	0.0398 (13)	0.0421 (11)	0.0015 (10)	0.0171 (9)	−0.0043 (10)
C8	0.0404 (10)	0.0444 (14)	0.0412 (11)	−0.0061 (10)	0.0089 (9)	0.0045 (11)
C9	0.0546 (13)	0.0496 (15)	0.0416 (11)	0.0159 (11)	0.0044 (9)	−0.0041 (11)
C10	0.0395 (10)	0.0320 (11)	0.0328 (10)	0.0023 (9)	0.0073 (8)	0.0002 (8)
C11	0.0349 (9)	0.0303 (11)	0.0326 (10)	−0.0006 (8)	0.0066 (8)	0.0007 (8)
C12	0.0548 (12)	0.0301 (11)	0.0323 (10)	0.0006 (10)	−0.0011 (9)	−0.0026 (9)
C13	0.0597 (15)	0.0450 (15)	0.0611 (15)	0.0176 (12)	−0.0013 (12)	−0.0005 (13)
C14	0.0349 (10)	0.0312 (11)	0.0323 (9)	0.0023 (8)	0.0051 (7)	0.0027 (8)
C15	0.0449 (12)	0.0502 (15)	0.0455 (12)	−0.0083 (11)	0.0206 (9)	−0.0110 (11)
C16	0.0403 (11)	0.0418 (14)	0.0507 (12)	−0.0106 (10)	0.0142 (9)	−0.0058 (11)
C17	0.0405 (10)	0.0285 (11)	0.0326 (9)	0.0027 (8)	0.0028 (7)	0.0002 (8)
C18	0.0430 (11)	0.0425 (13)	0.0348 (10)	−0.0046 (10)	0.0131 (8)	−0.0015 (10)
C19	0.0380 (10)	0.0416 (13)	0.0356 (10)	−0.0076 (9)	0.0099 (8)	−0.0011 (9)
C20	0.0684 (15)	0.0354 (13)	0.0610 (15)	0.0102 (12)	0.0003 (12)	−0.0033 (12)
N1	0.0405 (9)	0.0381 (11)	0.0339 (9)	0.0046 (8)	0.0082 (7)	−0.0027 (8)
O1	0.0568 (9)	0.0691 (13)	0.0514 (10)	−0.0151 (9)	−0.0030 (7)	−0.0109 (10)
O2	0.0398 (8)	0.0539 (11)	0.0615 (10)	0.0085 (8)	−0.0013 (7)	−0.0069 (9)
O3	0.0427 (7)	0.0306 (8)	0.0391 (7)	0.0057 (6)	0.0080 (6)	0.0016 (6)
O4	0.0711 (11)	0.0478 (11)	0.0528 (10)	−0.0009 (9)	0.0166 (8)	0.0124 (9)
O5	0.0465 (9)	0.0559 (12)	0.0697 (11)	−0.0093 (8)	0.0005 (8)	−0.0189 (9)
O6	0.0903 (13)	0.0467 (11)	0.0347 (8)	−0.0012 (10)	0.0100 (7)	−0.0013 (8)
S1	0.0475 (3)	0.0299 (2)	0.0381 (3)	0.0003 (2)	0.0016 (2)	−0.0037 (2)

### Geometric parameters (Å, º)

**Table:**

C1—C2	1.359 (5)	C11—C14	1.511 (3)
C1—C6	1.385 (3)	C11—H11	0.9800
C1—H1	0.9300	C12—O4	1.191 (3)
C2—C3	1.375 (5)	C12—O3	1.363 (3)
C2—H2	0.9300	C12—C13	1.492 (3)
C3—C4	1.391 (5)	C13—H13A	0.9600
C3—H3	0.9300	C13—H13B	0.9600
C4—C5	1.387 (4)	C13—H13C	0.9600
C4—H4	0.9300	C14—C19	1.385 (3)
C5—C6	1.379 (3)	C14—C15	1.393 (3)
C5—H5	0.9300	C15—C16	1.379 (3)
C6—C7	1.506 (3)	C15—H15	0.9300
C7—N1	1.461 (3)	C16—C17	1.383 (3)
C7—H7A	0.9700	C16—H16	0.9300
C7—H7B	0.9700	C17—C18	1.385 (3)
C8—O1	1.203 (3)	C17—S1	1.769 (2)
C8—N1	1.354 (3)	C18—C19	1.374 (3)
C8—O2	1.355 (3)	C18—H18	0.9300
C9—O2	1.421 (3)	C19—H19	0.9300
C9—C10	1.539 (3)	C20—S1	1.753 (3)
C9—H9A	0.9700	C20—H20A	0.9600
C9—H9B	0.9700	C20—H20B	0.9600
C10—N1	1.454 (3)	C20—H20C	0.9600
C10—C11	1.523 (3)	O5—S1	1.4353 (18)
C10—H10	0.9800	O6—S1	1.4346 (19)
C11—O3	1.445 (2)		
			
C2—C1—C6	121.0 (3)	O4—C12—O3	122.3 (2)
C2—C1—H1	119.5	O4—C12—C13	126.5 (2)
C6—C1—H1	119.5	O3—C12—C13	111.1 (2)
C1—C2—C3	121.1 (3)	C12—C13—H13A	109.5
C1—C2—H2	119.5	C12—C13—H13B	109.5
C3—C2—H2	119.5	H13A—C13—H13B	109.5
C2—C3—C4	118.7 (3)	C12—C13—H13C	109.5
C2—C3—H3	120.6	H13A—C13—H13C	109.5
C4—C3—H3	120.6	H13B—C13—H13C	109.5
C5—C4—C3	120.1 (3)	C19—C14—C15	118.6 (2)
C5—C4—H4	120.0	C19—C14—C11	121.46 (18)
C3—C4—H4	120.0	C15—C14—C11	119.88 (17)
C6—C5—C4	120.4 (3)	C16—C15—C14	120.81 (19)
C6—C5—H5	119.8	C16—C15—H15	119.6
C4—C5—H5	119.8	C14—C15—H15	119.6
C5—C6—C1	118.7 (2)	C15—C16—C17	119.47 (19)
C5—C6—C7	123.6 (2)	C15—C16—H16	120.3
C1—C6—C7	117.7 (2)	C17—C16—H16	120.3
N1—C7—C6	116.12 (18)	C16—C17—C18	120.39 (19)
N1—C7—H7A	108.3	C16—C17—S1	119.40 (16)
C6—C7—H7A	108.3	C18—C17—S1	120.20 (15)
N1—C7—H7B	108.3	C19—C18—C17	119.54 (18)
C6—C7—H7B	108.3	C19—C18—H18	120.2
H7A—C7—H7B	107.4	C17—C18—H18	120.2
O1—C8—N1	127.5 (2)	C18—C19—C14	121.09 (19)
O1—C8—O2	122.1 (2)	C18—C19—H19	119.5
N1—C8—O2	110.35 (19)	C14—C19—H19	119.5
O2—C9—C10	106.0 (2)	S1—C20—H20A	109.5
O2—C9—H9A	110.5	S1—C20—H20B	109.5
C10—C9—H9A	110.5	H20A—C20—H20B	109.5
O2—C9—H9B	110.5	S1—C20—H20C	109.5
C10—C9—H9B	110.5	H20A—C20—H20C	109.5
H9A—C9—H9B	108.7	H20B—C20—H20C	109.5
N1—C10—C11	113.76 (18)	C8—N1—C10	111.57 (18)
N1—C10—C9	101.58 (17)	C8—N1—C7	119.80 (18)
C11—C10—C9	110.60 (17)	C10—N1—C7	123.54 (17)
N1—C10—H10	110.2	C8—O2—C9	110.15 (17)
C11—C10—H10	110.2	C12—O3—C11	115.21 (16)
C9—C10—H10	110.2	O6—S1—O5	118.39 (12)
O3—C11—C14	108.86 (15)	O6—S1—C20	108.39 (13)
O3—C11—C10	106.93 (14)	O5—S1—C20	109.06 (13)
C14—C11—C10	112.74 (18)	O6—S1—C17	108.20 (11)
O3—C11—H11	109.4	O5—S1—C17	107.41 (11)
C14—C11—H11	109.4	C20—S1—C17	104.53 (11)
C10—C11—H11	109.4		
			
C6—C1—C2—C3	−0.5 (5)	C17—C18—C19—C14	1.3 (3)
C1—C2—C3—C4	0.0 (5)	C15—C14—C19—C18	1.4 (3)
C2—C3—C4—C5	0.2 (4)	C11—C14—C19—C18	−176.6 (2)
C3—C4—C5—C6	0.0 (4)	O1—C8—N1—C10	−174.6 (2)
C4—C5—C6—C1	−0.5 (3)	O2—C8—N1—C10	6.3 (2)
C4—C5—C6—C7	−177.8 (2)	O1—C8—N1—C7	−19.0 (3)
C2—C1—C6—C5	0.7 (4)	O2—C8—N1—C7	161.91 (19)
C2—C1—C6—C7	178.2 (3)	C11—C10—N1—C8	−122.93 (18)
C5—C6—C7—N1	−18.5 (3)	C9—C10—N1—C8	−4.1 (2)
C1—C6—C7—N1	164.21 (19)	C11—C10—N1—C7	82.5 (2)
O2—C9—C10—N1	0.6 (2)	C9—C10—N1—C7	−158.6 (2)
O2—C9—C10—C11	121.7 (2)	C6—C7—N1—C8	106.2 (2)
N1—C10—C11—O3	−64.6 (2)	C6—C7—N1—C10	−101.2 (2)
C9—C10—C11—O3	−178.14 (18)	O1—C8—O2—C9	175.1 (2)
N1—C10—C11—C14	175.82 (16)	N1—C8—O2—C9	−5.7 (3)
C9—C10—C11—C14	62.3 (2)	C10—C9—O2—C8	2.9 (3)
O3—C11—C14—C19	125.2 (2)	O4—C12—O3—C11	−3.3 (3)
C10—C11—C14—C19	−116.3 (2)	C13—C12—O3—C11	176.55 (18)
O3—C11—C14—C15	−52.7 (2)	C14—C11—O3—C12	−86.7 (2)
C10—C11—C14—C15	65.8 (2)	C10—C11—O3—C12	151.25 (17)
C19—C14—C15—C16	−2.5 (3)	C16—C17—S1—O6	139.88 (19)
C11—C14—C15—C16	175.5 (2)	C18—C17—S1—O6	−39.0 (2)
C14—C15—C16—C17	0.9 (4)	C16—C17—S1—O5	11.0 (2)
C15—C16—C17—C18	1.8 (3)	C18—C17—S1—O5	−167.86 (18)
C15—C16—C17—S1	−177.03 (19)	C16—C17—S1—C20	−104.8 (2)
C16—C17—C18—C19	−3.0 (3)	C18—C17—S1—C20	76.4 (2)
S1—C17—C18—C19	175.90 (18)		

### Hydrogen-bond geometry (Å, º)

**Table:**

*D*—H···*A*	*D*—H	H···*A*	*D*···*A*	*D*—H···*A*
C7—H7*A*···O4^i^	0.97	2.52	3.373 (3)	147
C10—H10···O6^i^	0.98	2.54	3.384 (3)	144
C13—H13*C*···O1^ii^	0.96	2.55	3.305 (3)	135

Symmetry codes: (i) *x*, *y*, *z*+1; (ii) *x*−1, *y*, *z*−1.
